# Functionalization of Polyethylene Terephthalate (PETE) Membranes for the Enhancement of Cellular Adhesion in Organ-on-a-Chip Devices

**DOI:** 10.1021/acsami.4c17706

**Published:** 2025-01-08

**Authors:** Carlos Sobejano de la Merced, Lavinia Doveri, Tomás Muñoz Santoro, Javier García, Junkal Garmendia, Iván Cortés Domínguez, Yuri Antonio Díaz Fernández, Carlos Ortiz de Solórzano

**Affiliations:** † 90212 University Clinic of Navarra Centre for Applied Medical Research, 31008 Pamplona, Spain; ‡ University of Navarra Clinic Cancer Center, 31008 Pamplona, Spain; § Inorganic Nanochemistry Lab, 19001 University of Pavia, 27100 Pavia, Italy; ∥ Instituto de Agrobiotecnología, 31192 Mutilva Baja, Spain; ⊥ CIBERES, 28029 Madrid, Spain; # CIBERONC, 28029 Madrid, Spain

**Keywords:** organ-on-a-chip, membranes, polyethylene terephthalate, cell adhesion, surface functionalization

## Abstract

Experimental reproducibility in organ-on-chip (OOC) devices is a challenging issue, mainly caused by cell adhesion problems, as OOC devices are made of bioinert materials not suitable for natural cellularization of their surfaces. To improve cell adhesion, several surface functionalization techniques have been proposed, among which the simple use of an intermediate layer of adsorbed proteins has become the preferred one by OOC users. This way, the cells use surface receptors to adhere to the adsorbed proteins, which are in turn attached to the surface. However, as protein adsorption is based on weak electrostatic bonding between the coating proteins and the substrate, this method produces suboptimal results: as the weak electrostatic bonds break, cells detach, leading to poor, heterogeneous cellularization. To solve this problem, we present a surface functionalization method for polyethylene terephthalate (PETE) membranes, commonly used in multilayer organ-on-chip devices to support cellular layers. This protocol involves hydrolyzation of the membrane, followed by (_3_-dimethylaminopropyl) carbodiimide (EDC) and *N*-hydroxysuccinimide (NHS) activation, resulting in covalent bonding between the membrane and coating proteins, much stronger than the weak electrostatic bonding provided by simple adsorption. As evaluation, we first measured the effect of the functionalization protocol in the morphological and mechanical integrity of the membranes. Next, we confirmed protein coating efficiency using the ζ potential and surface tension of the functionalized membranes coated with collagen type I, polylysine, gelatin, albumin, fetal bovine serum (FBS), and Matrigel. Finally, we showed that our method significantly improves the attachment of epithelial (A549) and endothelial (EA.hy926) cell lines under static conditions, especially in collagen-coated membranes, which were further tested under dynamic conditions, showing statistically significant improvement in cell attachment compared to uncoated or collagen-adsorbed only membranes.

## Graphical abstract

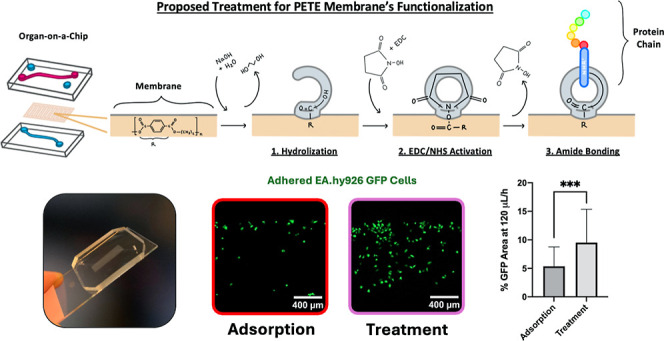

## 1 Introduction

Organ-on-a-chip (OOC) devices are microfluidic platforms consisting of cellularized channels, designed to model the structure, composition and physiology specific tissue organs. OOCs are promising preclinical models, as they can be used to study both normal and pathological processes with a high degree of accuracy and reasonable cost, compared to the alternative standard cell culture systems or to the use of animal models, respectively. Notably, on December 29, 2022, the U.S. Food and Drug Administration (FDA) officially recognized OOC devices as a complementary option for nonclinical testing.^1^ OOCs can be made of one single cellularized layer, as in those OOCs used to simulate, for instance, the endothelial wall, or the epithelial layer of a given tissue. OOCs can also contain two or more micropatterned cellularized layers separated by porous interfaces. These multilayer OOCs can more physiologically mimic the three-dimensional architecture of organs, including the interactions between different cell layers, and the existence of interstitial flows between them.

OOCs are typically fabricated using cost-effective materials. The materials most commonly used for the enclosure and the micropatterned channels are glass, polydimethylsiloxane (PDMS), polystyrene (PS), poly­(methyl methacrylate) (PMMA), polycarbonate (PC) or cyclic olefin copolymer (COC).^2^ The porous interfaces surfaces that separate the channels in multilayered OOCs are usually made of PDMS, silica, poly­(lactic-*co*-glycolic acid) (PLGA), collagen, polyethylene terephthalate (PETE), Teflon (PTFE) or PC.^3^ Among these materials, PETE, a polyester synthesized via the polycondensation of terephthalic acid and ethylene glycol, offers several advantages: PETE membranes are inexpensive, biocompatible, mechanically and chemically resistant porous surfaces that facilitate optimal cellular proliferation and efficient media exchange between device channels. Moreover, PETE membranes are commercially available in a wide range of pore sizes, are transparent and easily modifiable compared to other materials, which make them a material of choice for OOC devices.^4–6^

Despite their potential, there are still challenging issues that need to be addressed to improve OOC’s, their low reproducibility being the most important one.^7^ Specifically, one of the most critical, unsolved issues for the effective use of OOC devices is the correct cellularization of the channels and porous interfaces, which rely on the strength of the interactions between the cells and the substrate material. These interactions start with cell attraction to the surface mediated by electrostatic or van der Waals forces. Cells then adhere to the surface through adhesion receptors such as integrins and cadherins, forming labile interactions with the available surface ligands, native to the original substrate or artificially generated through physiochemical modifications. Finally, the cell membrane receptors cluster and form focal adhesion complexes which can mature, contributing to stronger cell adhesion.^8^

As OOC surfaces are usually made of bioinert materials, surface functionalization is required to enhance cell adhesion. To this end, some authors propose preadsorption of proteins to the target surface, with the aim to enhance the interactions between the surface and the cells.^9–11^ The most commonly used proteins are extracellular matrix proteins for which cells have high affinity. This has become the standard method due to its simplicity. However, the bonds produced this way between the surfaces and the proteins are usually too weak to handle the shear stress caused by the high flows that exist within the OOC’s microfluidic channels, resulting in cell detachment. This often leads to limiting the flows that can be used within the devices, restricting the extent of their potential application. For these reasons, it is crucial to develop robust, reliable surface coating protocols to improve cell adhesion to the OOC’s surfaces.

Protein immobilization is a promising technique designed to improve cellularization of OOC microfluidic devices. Among protein immobilization methods, alternative plasma surface treatments such as atmospheric pressure plasma jet (APPJ) and plasma-activated coating (PAC) have gained recent attention due to their capacity to bond proteins covalently without the need for additional reagents.^12,13^ APPJ involves electrically ionizing gases at atmospheric pressure to generate a plasma plume, which can modify surface properties. PAC uses energized ions bombarded onto surfaces under a negative bias, enabling the incorporation of functional groups. Both methods introduce reactive groups, such as carbonyl and carboxylic groups, which can subsequently interact with coating proteins.^12,13^ However, these techniques presents certain limitations. APPJ can be overly aggressive for thin membranes, potentially causing surface ablation.^13^ In addition, it requires an assisted scanning system to achieve uniformly treated surfaces, which adds complexity and cost of the method. PAC, on the other hand, typically involves high deposition temperatures, rendering it unsuitable for temperature-sensitive substrates.^14^ Finally, the equipment required for both APPJ and PAC is expensive, limiting their accessibility in many laboratories.

To address the mentioned limitations and demands, this work presents a simple, robust and cost-effective surface treatment method aimed at improving cellular adhesion to the porous interfaces of multilayered OOC devices. Specifically, we present and characterize a two-step protocol for the functionalization of PETE porous membranes (Scheme 1). The first step of the protocol increases the number of reactive sites at the membrane, via controlled hydrolyzation with sodium hydroxide (NaOH). This induces a controlled, partial cleavage of intramolecular PETE bonds, leading to an increase of exposed carboxylic (COOH) groups on the surface, which enhance protein attachment to the surface, as has been previously shown in the textile industry.^15^ The second step generates covalent bonds between the coating proteins and the surfaces through the activation of the COOH groups. This activation process, previously used to functionalize polylactic acid nanoparticles,^16^ exploits (3-dimethylaminopropyl) carbodiimide (EDC) and *N*-hydroxysuccinimide (NHS) chemistry, leaving a more reactive ester group at the surface. This way, amide bonding can be enhanced between the coating proteins and the surface of the OOC device.

**Scheme 1:**
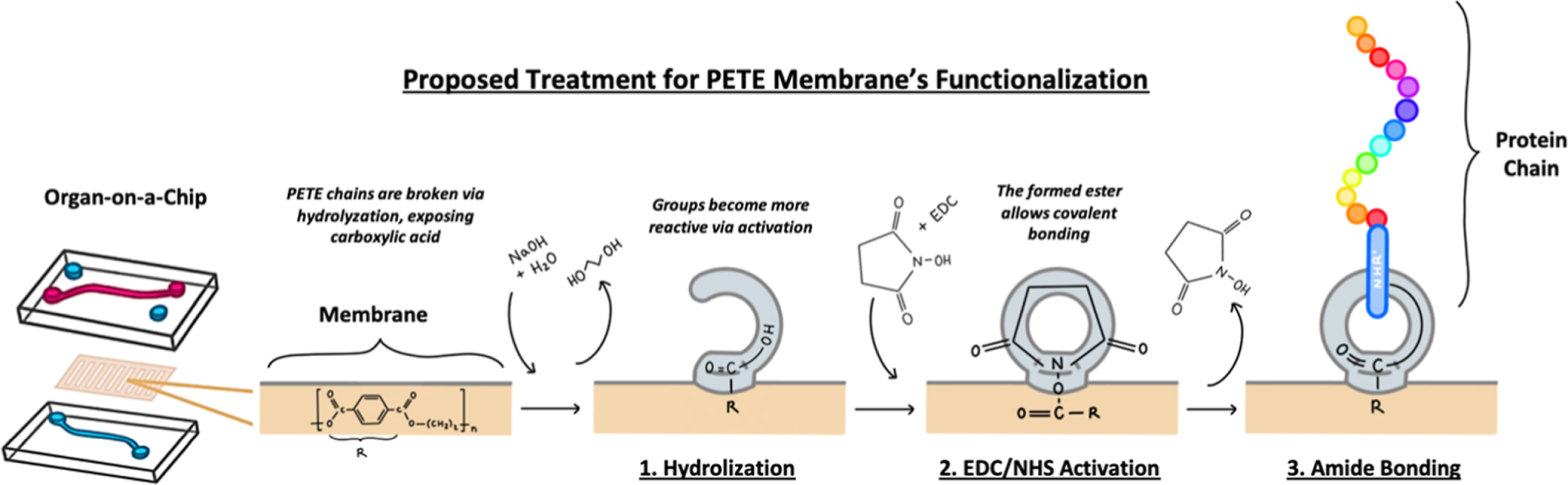
Schematic of the Proposed Protocol for Functionalization of PETE Membranes^a^

a 1. Membranes undergo controlled hydrolyzation, causing a major exposure of COOH groups; 2. Then, the COOH groups are activated via EDC/NHS; 3. Allowing covalent bonds between the protein chain and the surface.

The concentration of NaOH used during the hydrolyzation step was optimized, and the resulting surfaces were characterized by complementary surface analysis techniques: scanning electron microscopy (SEM), ultraviolet–visible (UV–vis) spectroscopy, and mechanical traction assays to test both the morphology and mechanical integrity of the surfaces. We also measured the chemical properties of the membranes coated with different proteins, activating and nonactivating the carboxylated surfaces with EDC/NHS. To this end, the surface ζ potential and contact angle was measured to determine the dispersive and polar components of the surface tension, which can be related to the extent and mechanism of protein coating.

Finally, as a proof of concept, we tested the effect of our protocol in the adhesion of lung epithelial (A549) and vascular endothelial (EA.hy926) cell lines to PETE membranes acting as interchannel porous interfaces in multilayer airway-on-a-chip devices that simulate the terminal ends of the lung lower airways. Cell adhesion experiments were performed under static and dynamic flow conditions, using the same micropatterned channels used in the OOC devices.^10^

## 2 Materials and Methods

### 2.1 Preparation of Carboxylated PETE Membranes

PETE porous membranes (product code 1300018) were purchased from STERLITECH (Auburn, WA). The membranes were cut using a scalpel and then cleaned using 70% ethanol and deionized (DI) water. Next, the membranes underwent controlled hydrolyzation using 2 mL NaOH solutions at different concentrations (0.25, 0.5 and 1 M) at 60 °C for 4 h. Finally, the PETE pieces were rinsed with DI water and dried at room temperature (RT).

### 2.2 Analysis of Hydroxylic Group Formation by Malachite Green Test

Hydrolyzed membranes were stained with 0.01 M malachite green solution in 0.1 M acetate buffer at pH 5.0. The membranes were shaken at 160 rpm for 1 h at RT. The excess of stain was removed by washing the membranes three times with clean DI water. The absorbance profile of the different samples was measured using a UV–vis spectrophotometer (UV–vis Cary 60, Agilent, CA, USA) scanning wavelengths from 1100 to 200 nm, with the aim of analyzing the absorbance peak produced by the adhered malachite green. To analyze the absorbance profiles, an algorithm was developed using Matlab R2022b, that is described in Section S1 and Scheme S1.

### 2.3 Membrane Porosity and Integrity Using SEM

Square membrane pieces (1 cm × 1 cm) were inserted in SEM stubs using carbon tape. Samples were prepared for SEM microscopy via sputtering (Polaron E5000C, AGAR), covering the surface with gold in 3 cycles of 30 s. Finally, the samples were imaged under a microscope (Zeiss EVO MA10, Carl Zeiss, Oberkochen Germany) to visualize the porous structure of the treated membrane. Eleven images were taken from each sample with 5000× and 2000× magnifications, under an electron high tension (EHT) of 5 kV. The acquired SEM micrographs were analyzed using an algorithm developed in Fiji (ImageJ)^17^ and described in Section S1. The goal of the algorithm was to segment all the pores in the images and calculate their circularity, their diameter, the porosity of each sample, measured as the ratio of the number of pores to the total area analyzed (pores cm^–2^), and the percentage of available bulk membrane, estimated by subtracting the sum of all pores’ areas from the total area analyzed, and dividing the result by the total area analyzed.

### 2.4 Tensile Test Analysis

First, the thickness of the treated membranes was measured using a thickness gauge. After that, the mechanical properties of the resulting surfaces were assessed using a universal tensile machine (TA.XT Texture Analyzer testing machine, Stable Micro Systems, Godalming, UK), equipped with a 5 kg load cell and a special adapter for film testing. Briefly, 3 cm × 1 cm membranes were held vertically using two mechanical clamps and a controlled stress was applied until the membranes broke, while recording the force-strain behavior of the films. The collected data was used to determine the tensile strength, the maximum elongation of the membranes and their Young’s modulus. To obtain these data, a program was developed in Matlab R2022b. First, recorded force (mN) and elongation (μm) were expressed in terms of stress (Pa) and deformation (%) to build the stress deformation plots. The maximum elongation can be obtained by selecting the elongation associated with the maximum force value, which is the tensile strength. The Young’s modulus was calculated by determining the slope of the linear region of the graph.

### 2.5 EDC/NHS Activation of Carboxylated Membranes

EDC (03450-5G, Merck) and NHS (130672, Merck) solutions were prepared by dissolving the powdered reagents in 0.1 M 2-(*N*-morpholino) ethanesulfonic acid (MES) buffer (pH 5.5) at a concentration of 1 mg mL^–1^. The process began with the immersion of the membranes in 1 mL of EDC solution, followed by the addition of the same proportion of the NHS solution (2 mL final volume). The samples were then maintained at 4 °C for 1 h to allow the reaction to occur on the surface of the membranes.

### 2.6 Coating of Proteins on Carboxylated PETE Membranes

Carboxylated membranes, either pre- or post-EDC/NHS activation were washed with DI water and exposed to different protein solutions at a concentration of 0.1 mg mL^–1^ in 0.01 M carbonate buffer (pH 8.4). The proteins used were rat tail type I collagen (11563550, Corning), poly-l-lysine (P8920, Merck), gelatin from porcine skin (G1890, Merck), human albumin (A1653, Merck), HyCloneTM FetalCloneTM III serum (10570083, Cytiva) and Matrigel (45356231, Corning). Samples were maintained in the solutions for 1 h at RT in an orbital shaker at 180 rpm. For the Matrigel solution, to avoid solidification, the treatment was performed at 4 °C. After this, the membranes were rinsed with DI water three times and left to dry.

### 2.7 Direct Adsorption of Collagen on PETE Membranes

PETE membranes washed in 70% ethanol and DI water were immersed in 2 mL of type I collagen (11563550, Corning) diluted to 0.1 mg mL^–1^ in a 0.02 M acetic acid solution. The membranes were then incubated at 37 °C for 2 h to let the adsorption occur. Subsequently, the surfaces were washed three times with DI water.

### 2.8 ζ Potential Analysis for Surface Charge Characterization

The surface electric potential of the membranes was characterized using a surface ζ potential cuvette (Malvern Zen1020) on a Malvern Zetasizer DLS system (Zetasizer nano-ZS90, Malvern, UK). Rectangular membrane samples (7 mm × 4 mm) were fixed in a sample holder using double-sided tape. The sample holder was then placed in the surface ζ potential cell and immersed in a 50-particles per million (ppm) tracer solution in 1% (v/v) phosphate-buffered saline solution (PBS) to perform measurements at pH 7. Depending on the surface charge, positively charged (NR3+) or negatively charged (SO_3_H) nanoparticles (Micromer) were used as tracers. Measurements were conducted following the manufacturer’s instructions, with three measurements taken at five different positions.

### 2.9 Contact Angle Measurements for Determining the Surface Tension

Surface tension components were measured using a contact angle goniometer (KSV CAM200, KSV Instruments, Finland). Drops of 20 μL of different solutions (water, PBS, and glycerol) were deposited on each modified surface to assess the polar and dispersive components of surface tension. Contact angle measurements were obtained by capturing 20 images after deposition. The components of the surface tension were calculated using the Owens–Wendt–Rabel–Kaelble formula based on the obtained results.

### 2.10 Cell Culture and Maintenance

A549 human male alveolar basal epithelial (CCL-185, ATCC, LGC-Promochem, Barcelona, Spain) and EA.hy926 human umbilical vein endothelial cell lines (CRL-2922, ATCC, LGC-Promochem, Barcelona, Spain) were used. Both cell lines were stably transfected with plasmids for lentiviral expression of enhanced green fluorescent protein (pLEGFP) (donated by Dr. Serrano, CNIO, Madrid, Spain) using the X-tremeGENE 9 DNA transfection kit (Roche, Mannheim, Germany) following manufacturer’s protocol. Clones were selected by culturing cells in the presence of 1 mg mL^–1^ G418, obtaining fluorescent cell lines as a result. Cells were thawed from frozen stock in 50 mL Falcon tubes containing 30 mL of Dulbecco’s modified Eagle medium (DMEM) (41966029, Gibco, Spain) and centrifuged to remove traces of cryoprotectant dimethyl sulfoxide (DMSO). They were then incubated in T75 flasks with 10 mL of DMEM medium supplemented with 10% FBS (10570083, Hyclone, USA) and 1% Penicillin/Streptomycin (Pen/Strep) (15140122, Gibco, Spain) at 37 °C and 5% CO_2_. Upon reaching 90% confluence, cells were subcultured into new T75 flask using 0.05% trypsin-ethylenediaminetetraacetic acid (EDTA) (25200056, Gibco, Spain) followed by centrifugation, resuspension in the described fresh supplemented DMEM media, and subsequent incubation under standard conditions (37 °C, 5% CO_2_).

### 2.11 Cellular Adhesion Assay on the Treated Surfaces under Static Conditions

These experiments were conducted in glass-bottom 24-well plates using built-in microfluidic devices inserted into the wells to fix the position of the membranes to the bottom of the plate, ensuring an equal surface area of exposed membrane in all wells. The insert-device (Figure S1) consists of three elements: a 13 mm diameter ring of PDMS with an inner hole of 6 mm diameter, a treated PETE membrane, and a 13 mm diameter 0.17 mm thick glass coverslip. The PDMS ring and the coverslip are covalently attached by a 1 min oxygen plasma treatment at 85 W (Diener Zepto, Germany) thus firmly fixing the membrane in between. The insert-devices were firmly attached to the bottom of the well by putting a drop of uncured 10:1 PDMS-curing agent on the coverslip side of the device. The devices were stored at 37 °C overnight to allow the PDMS to cure without altering the proteins on the membrane. The customized well plates loaded with the insert-devices were sterilized using 70% ethanol and exposed to UV light for 15 min. Next, the wells were cleaned using sterile Milli-Q water and dried at RT. Then, 50 μL of a cell suspension at 120,000 cell mL^–1^ was pipetted on the center of each ring. The plates were then stored in the incubator at 37 °C and 5% CO_2_ for 30 min to allow the cells settle on the surface of the membranes. After this incubation period, 1 mL of complete DMEM medium was poured on each well, and the devices were placed again in the incubator for 4 h to let the cells adhere to the surfaces. Finally, the wells were washed twice with PBS and refilled again with 1 mL of fresh DMEM medium. The area occupied by the membrane within each well, covered by cells, was imaged under a widefield Zeiss Cell Observer Z1 fluorescence microscope by exciting the cells at 493 nm and filtering the obtained signal with a 500–550 nm emission filter. Tiles of images (10,525 × 10,801 pixels) were acquired covering each membrane sample using a 10× objective (Zeiss *N*-ACHROPLAN 10×/0.25 Ph1, ∞/-) and an Axiocam MRM camera (Zeiss). The acquired image tiles were analyzed using a custom-made plugin developed in Fiji/ImageJ to calculate the percentage of the area of each membrane covered by cells inside the PDMS ring.^17^ The analysis was conducted by the Imaging Platform at the Centre for Applied Medical Research (CIMA-University of Navarra, Pamplona, Navarra, Spain). The macro was designed to automatically detect the device edges, identify green fluorescent protein (GFP)—positive cells, and measure the area fraction between GFP-positive cells and the total device area. GFP-expressing cells were segmented by first removing the background signal using a rolling ball algorithm, followed by local contrast enhancement using contrast-limited adaptive histogram equalization (CLAHE) and a median filter. Rolling ball algorithm determines the local background value for every pixel by averaging over a large ball around the pixel, which is then subtracted from the original image to remove large spatial variations of the background intensities. To delimit the GFP-positive phenotype, Otsu’s automatic thresholding method was used.^18^ Otsu’s method analyzes all possible thresholds to divide images in two groups, and optimizes the division by choosing the threshold for which the sum of the intragroup variances is minimized.

### 2.12 Cellular Adhesion Assay on the Treated Surfaces under Dynamic Conditions

Customized microfluidic devices were fabricated to test cell adhesion under dynamic conditions. These devices were designed to replicate the dimensions and geometry of either the endothelial or the epithelial cell compartments of typical multilayer airway-on-a-chip devices.^10^ The devices consist of three layers: a PDMS slab with the micropatterned channel, the membrane where the cells will attach, and a glass slide that seals the device (Figure S2). The PDMS channel slabs were fabricated using replica-molding techniques. The mold used to replicate the 1 mm width × 1 mm height epithelial channel was fabricated via stereolithography in a Form 2 3D printer (Formlabs) using Tough 2000 V2 material. The mold for the 0.2 mm width × 1 mm height endothelial channel consisted of a patterned silica wafer fabricated by photolithography using SU8-2100 resin (Kayaku, MA, USA). For more details, see Section S1. Both channels are 10 mm long. To fabricate the patterned slabs, a 10:1 PDMS-curing agent mixture was added to the molds to obtain a thickness of approximately 4 mm. Then, the casted objects were left in an oven at 65 °C to let the polymer cure. Once hardened, the PDMS slabs were detached and cut with a scalpel. Finally, to seal the devices, rectangular glass slides were adhered covalently to the patterned surfaces via oxygen plasma bonding (30 s, 85 W), while embedding in the PETE membrane in the process. The fabricated devices were first sterilized using 70% ethanol and exposing them to UV light for 15 min. Next, the devices were washed with sterile Milli-Q water. The devices replicating epithelial compartments were filled with a solution of 120,000 A549 GFP cells mL^–1^ in complete DMEM, letting the cells attach to the surface for 4 h in the incubator at 37 °C. The devices replicating the endothelial channels were seeded with a solution of 600,000 EA.hy926 GFP cells mL^–1^ to have the same number of cells in both channels. After the incubation period, the devices were connected to a 1 mL syringe pump via 24 G PTFE tubing and perfused with fresh medium at various flow rates for 1 h. The lowest flow rates applied were comparable in magnitude to those reported in similar experimental setups described in the literature.^10,12^ Increased, moderate flow and high flows were used to test the protocol in increasingly demanding situations, as described in the Section 3. A summary of the flow regimes used for each cell line and the corresponding shear stresses is presented in Table 1.

**Table 1:** Flow Rates and Shear Stresses Applied to Different Cell Types to Compare the Effectiveness of the Different Treatments

cell type	low flow rate		moderate flow rate		high flow rate	
	flow (μL h^–1^)	shear stress (dyn cm^–2^)	flow (μL h^–1^)	shear stress (dyn cm^–2^)	flow (μL h^–1^)	shear stress (dyn cm^–2^)
A549	50	0.0008	200	0.0032	800	0.013
EA.hy926	30	0.012	120	0.048	480	0.192

Once completed the hour of flow, the microfluidic channels were unplugged from the pump and were imaged under a widefield fluorescence microscope (Zeiss Cell Observer Z1) to determine the area of the channel covered by cells. As in the static experiments, the rectangular region of each channel, containing the membrane, was captured with a Zeiss *N*-ACHROPLAN 10× objective (0.25 Ph1, ∞/-) and an Axiocam MRM camera (Zeiss), obtaining images of 23,128 × 3420 pixels. The fluorescence was excited through a 493 nm and the emitted light was filtered with a 500–550 nm filter. Each tile was divided into 8 images of squared fields of view of approximately 1 mm^2^. The obtained images were analyzed with a plugin in Fiji/ImageJ to determine the density of cells in the channels, which was also developed by the Imaging Platform at the Centre for Applied Medical Research (CIMA-University of Navarra, Pamplona, Navarra, Spain).^17^ The macro was designed to perform an automatic segmentation of the linear section of the channel, identify GFP-positive cells, and measure the area fraction of GFP signal referenced to the total channel surface area. GFP cells were segmented by first removing the background signal using the rolling ball algorithm. Then, a local contrast enhancement is applied, using CLAHE and a median filter. To define GFP-positive phenotype classification, Otsu’s automatic thresholding method was used.^18^

### 2.13 Monolayer Formation/Tight Junctions Assay

Microfluidic devices described in the previous paragraph were used to assess the monolayer formation using the proposed treatment with type I collagen. In this case, the devices were sealed with a coverslip instead of a glass slide to improve the microscopy acquisition. The fabricated devices were sterilized and washed following the guidelines given previously. The devices replicating epithelial compartments were filled with a solution of 500,000 A549 GFP cells mL^–1^ in complete DMEM, letting the cells attach to the surface for 4 h in the incubator at 37 °C. The devices replicating the endothelial channels were seeded with a solution of 2,500,000 EA.hy926 GFP cells mL^–1^ to have the same number of cells in both channels. After incubation, the devices were connected to a 10 mL syringe pump through 24 G PTFE tubing, for 2 days for the epithelial channel and three for the endothelial one, due to differences in the proliferation rate of each cell line. Moderate flows of 200 and 120 μL h^–1^ (see Table 1) were used for epithelial and endothelial channels, respectively. Next, the devices were disconnected from the syringe pump and fixed using 4% formaldehyde for 15 min at 37 °C. The devices were cleaned by flowing PBS 1× through the channels three times. Then, the devices were blocked using 1% bovine serum albumin (BSA) (w/w) in PBS for 15 min and stained using 1:10 anti human E-cadherin conjugated with Alexa Fluor 555 (clone 67A4, lot number 2077969, product code 560064, BD Biosciences) or 1:10 anti human platelet endothelial cell adhesion molecule (PECAM-1) conjugated with Alexa Fluor 594 (clone WM59, lot number B363385, product code 303126, Biolegend) in 1% BSA solution. Samples were incubated at RT for 2 h in the dark. Finally, samples were cleaned using PBS 1× three times. Once prepared, the devices were observed using a confocal microscope (Zeiss LSM 800). High resolution images of 319.45 μm × 319.45 μm were obtained using a Zeiss Plan-Neofluar 40× objective (AN 1.30, Oil). The fluorescence was excited with a 553 nm-wavelength laser. For E-Cadherin detection, emitted fluorescence in the 550–700 nm range was captured. For PECAM-1, light between 580 and 700 nm was collected.

### 2.14 Statistical Analyses

Statistical analyses were performed using Prism software, version 8 for Mac (GraphPad Software) and are detailed in each figure legend. Results were reported as mean ± standard deviation (SD) or median ± range. Where appropriate regarding to the normality and standard deviations of analyzed samples, statistics were calculated using unpaired two-tailed *t* tests, and Welch tests. Also, ANOVAs (ordinary one-way ANOVA, Brown-Forsythe and Welch ANOVA tests) and Kruskal–Wallis tests were performed in some studies with their respective posthoc analyses. In all cases, *P* < 0.05 values were considered statistically significant (*P ≤ 0.05, **P ≤ 0.01, ***P ≤ 0.001, ****P ≤ 0.0001.).

## 3 Results and Discussion

### 3.1 Effective Hydrolyzation of PETE Membranes. Analysis of the Effect in the Morphological and Mechanical Properties of the Membranes

We hypothesized that the generation of COOH groups at the PETE membrane surface should improve reactivity toward surrounding biomolecules. With this in mind, we performed controlled hydrolyzation of PETE membranes using different concentrations of NaOH. The extent of surface hydrolyzation was quantified by the malachite green test to measure the abundance of COOH groups at the surface, exploiting the interactions between them and this organic dye. The intensity of malachite green absorbance, corrected to the baseline absorbance spectrum, was measured for control and treated membranes. The complete absorbance peak and baseline data are available in the Supporting Information file (Tables S1 and S2) and the main results are summarized in Figure 1. The exposure of the PETE membranes to NaOH results in an increase in malachite green absorbance (Figure 1A), confirming the increased availability of COOH groups on the treated surfaces. Our results also show that excessive hydrolyzation reduces the effectiveness of the treatment, possibly due to the degradation of the membrane material. This is confirmed by the increase in baseline absorbance observed on hydrolyzed samples for increasing NaOH concentrations (Figure 1B), which can be attributed to etching occurring during the treatment. Indeed, as the treatment becomes more aggressive, i.e. under higher concentrations of alkaline solution, the membranes become darker due to PETE fiber degradation (Figure 1C). Therefore, the optimal treatment is 0.25 M NaOH, as it provides the best surface activation with minimal degradation of the material.

**Figure 1:**
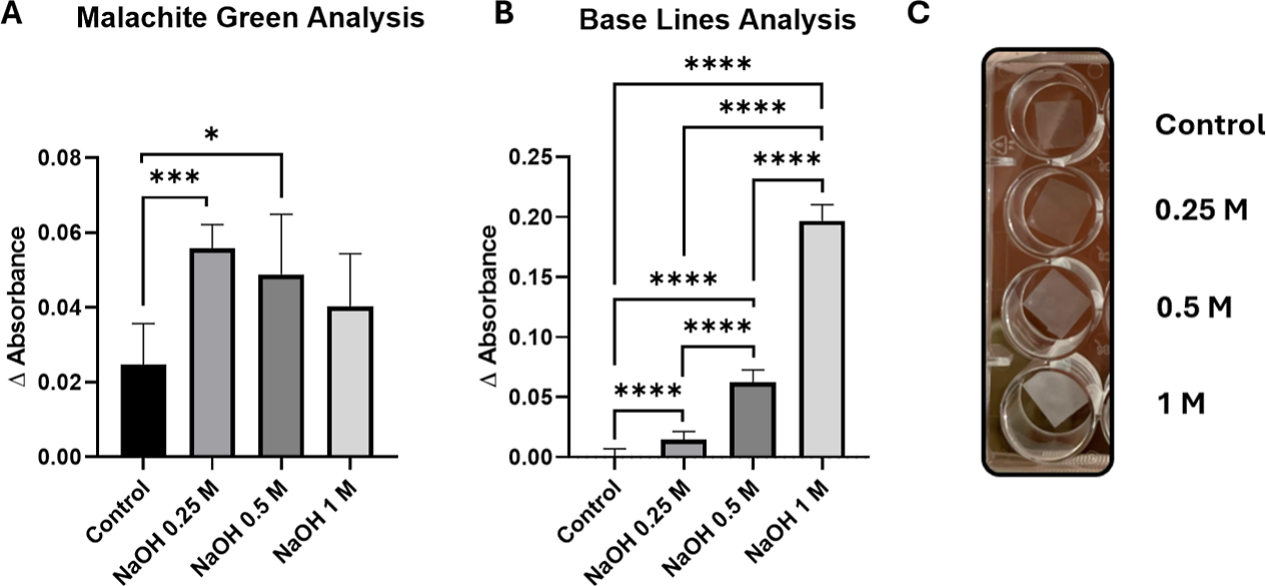
Malachite green test of hydrolyzed PETE membranes. (A) Absorbance of malachite green on hydrolyzed samples at different NaOH concentrations (λ_max_ = 640 nm), related to the number of COOH available groups. (B) Baseline absorbance (520–750 nm range) of hydrolyzed samples. (C) Image showing the opacity of the hydrolyzed membranes as the concentration of NaOH increases. Data represented in (A) is the median ± range; *n* = 6 membranes (Kruskal–Wallis test, Dunn’s posthoc test). Data represented in (B) is the media ± SD; *n* = 6 membranes (ordinary one-way ANOVA test, Tukey’s posthoc test).

Next, we analyzed the effect of hydrolyzation on the integrity of the PETE membranes by measuring changes in pore morphology using SEM microscopy. Figure 2 shows a summary of the results. As anticipated, increasing NaOH concentrations resulted in larger (Figure 2A) and more irregularly shaped pores (Figure 2B). At 0.25 M NaOH, the pore morphology was minimally affected compared to higher NaOH concentrations, while maintaining porosity levels comparable to the untreated controls (Figure 2C). At elevated NaOH concentrations, the increased etching of the material led to a significant reduction in membrane volume and structural availability (Figure 2D). However, at 0.25 M, the observed material loss was negligible relative to the controls. These morphological differences are visually apparent in the representative SEM images shown in Figure 2E.

**Figure 2:**
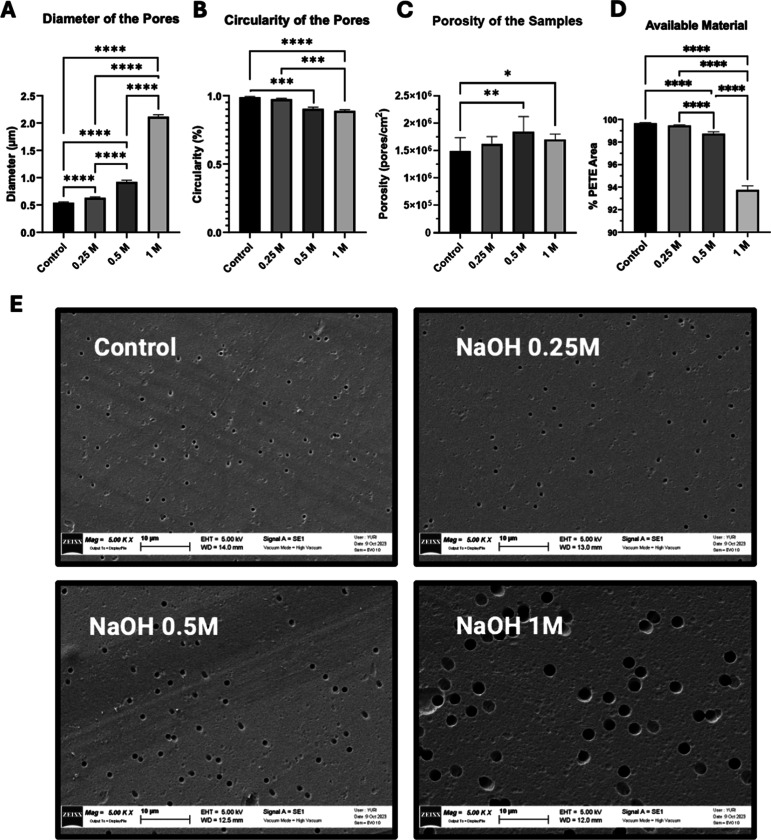
Morphological characterization of hydrolyzed PETE surfaces. (A) Pore diameter of the hydrolyzed samples, (B) circularity of the pores in the treated membranes, (C) porosity of the membranes and (D) available membrane area for increasing NaOH concentrations. Data represented in (A–D) is the mean ± SD; *n* = 11 fields of view (FOV) (1 membrane, 11 images) [(A,D) Ordinary one-way ANOVA, (B) Kruskal–Wallis and (C) Brown–Forsythe and Welch tests; Tukey’s, Dunn’s and Games–Howell posthoc tests respectively]. (E) Treated membranes under SEM microscopy, at a 5000× magnification.

Finally, we analyzed the mechanical properties of the hydrolyzed membranes: the Young’s modulus that quantifies the ability of a material to resist deformation under an applied load and is a measurement of the stiffness of the material; the tensile strength, which represents the maximum amount of mechanical stress that a material can withstand before rupture; and the maximum elongation. Our results, shown in Figure 3, suggest that the key mechanical properties of the membranes treated with 0.25 M NaOH remained practically unaltered, as the changes in Young’s modulus (Figure 3A) and maximum elongation (Figure 3C) are not significant compared to the untreated control membranes. Interestingly, the maximum stress that the membranes can withstand increases at this low concentration of NaOH (Figure 3B), which also leads to a slightly higher elongation capacity of the membrane. This may be attributed to the interactions among the newly generated carboxylic groups, including van der Waals forces and hydrogen bonds. Due to these interactions, the stress required to displace the molecules becomes higher, resulting in a stronger surface. At higher concentrations of NaOH however, membrane characteristics change significantly: the Young’s modulus increases, and the enhanced tensile strength and maximum elongation decrease, altering significantly the mechanical properties of the original substrate.

**Figure 3:**
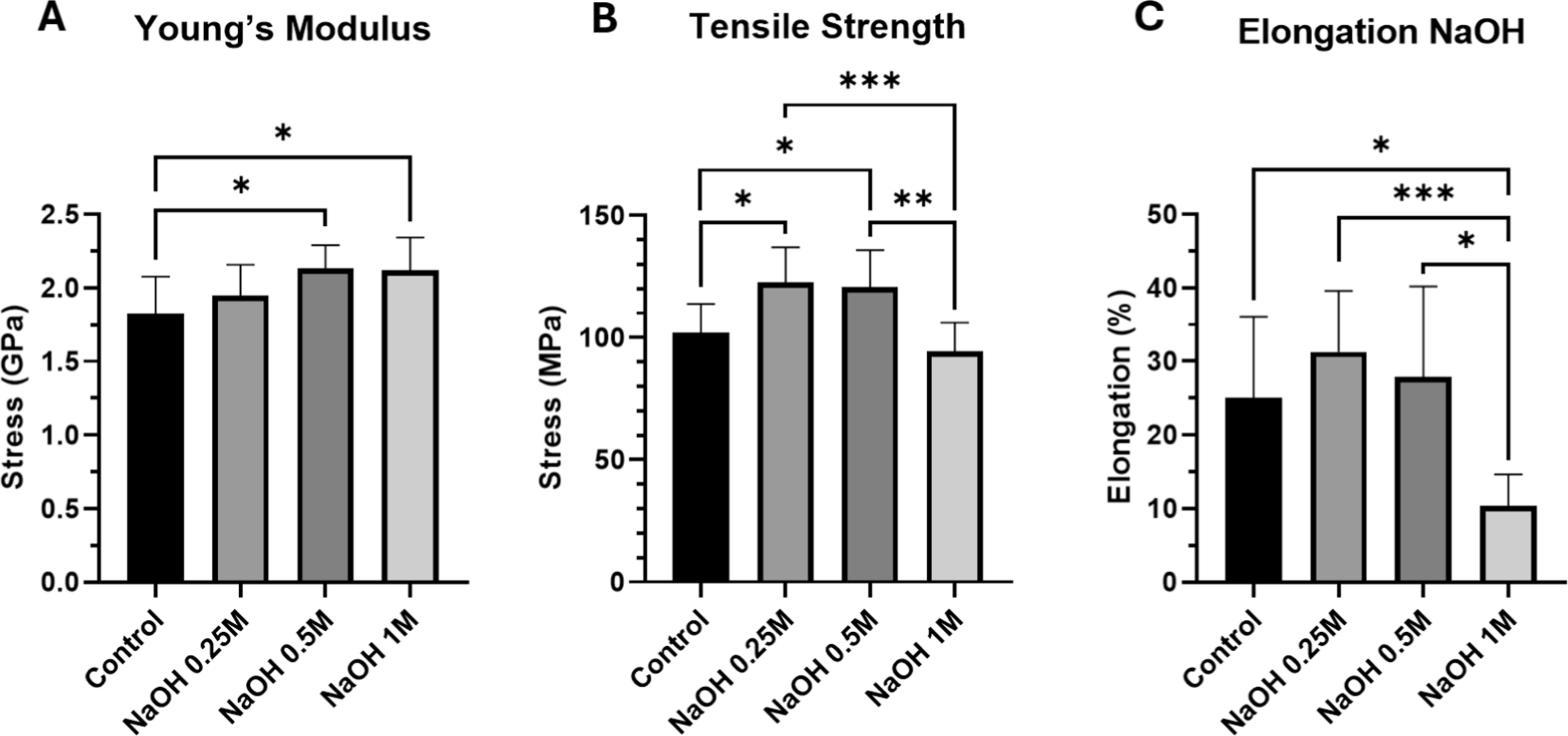
Mechanical characterization of hydrolyzed PETE surfaces. (A) Young’s modulus for increasing NaOH concentrations. (B) Changes in the tensile strength for increasing NaOH concentrations. (C) The elongation capacity of the membranes for increasing NaOH concentrations. All the graphics represent the media ± SD; *n* = 9 membranes [(A,B) ordinary one-way ANOVA with Tukey’s posthoc tests, (C) Brown–Forsythe and Welch ANOVA tests with Games–Howell’s posthoc test].

Taking our combined morphological and mechanical characterization data into account, we confirm that the treatment at concentration 0.25 M NaOH for 4 h at 60 °C is optimal for exposing a high number of COOH groups on the surface, while minimally altering the physical properties of the membrane. This optimal treatment was therefore used before further functionalization steps.

### 3.2 Chemical Characterization of Protein-Coated Surfaces

Hydrolyzed PETE membranes with and without EDC/NHS activation were coated with different proteins: human albumin, gelatin, poly-l-lysine, type I collagen, FBS or Matrigel. Protein-coated membranes were next characterized to analyze the extent and stability of the protein coatings. ζ potential measurements were carried out to compare the electric charge of the substrates before (Control) and after protein coating. The results obtained are displayed in Figure 4. Please note that FBS and Matrigel surfaces were not characterized with this technique, as the nanoparticle tracers used to measure the surface potential exhibited abnormal behavior during the measurements. This can be due to the fact that these are complex samples containing not only adhesion-related proteins but also enzymes, growth factors, and other nonadhesion-related proteins that affect the stability of the nanoparticles. Figure 4 shows that control, uncoated membranes have a negatively charged surface, likely due to the generation of carboxylic groups at the surface, thus confirming the effectiveness of the hydrolyzation.

**Figure 4:**
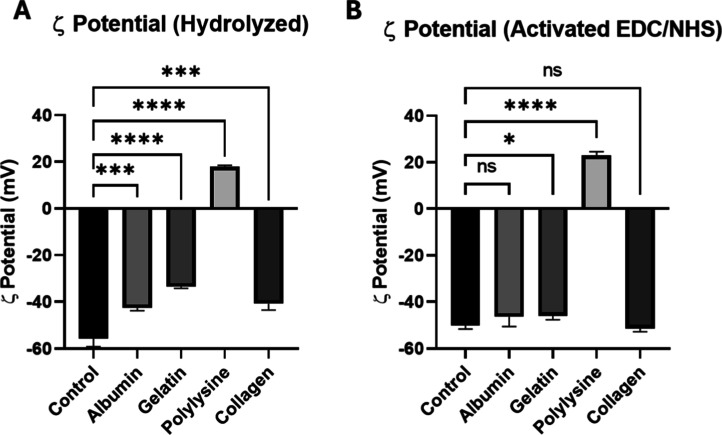
ζ potential analysis of protein coated surfaces. ζ potential measurements at physiological pH for (A) hydrolyzed-only nonactivated surfaces and (B) hydrolyzed and EDC/NHS activated surfaces. The graph represents the media ± SD; *n* = 3 membranes (unpaired two-tailed *t* tests).

Next, to rationalize the different behavior observed for the specific proteins, we analyze the ζ potential results considering their isoelectric points, listed in Table 2. Overall, the ζ potentials of all the hydrolyzed, nonactivated, protein coated surfaces are less negative than the control, untreated surface (Figure 4A), indicating a change in the surface charge caused by the successful coating with the proteins. In the activated surfaces (Figure 4B), by contrast, the differences of ζ potential values are smaller or not significant relative to the control, except for polylysine-coated surface. The high positive charge of polylysine molecules explains the stronger electrostatic interaction between this protein and the negatively charged hydrolyzed surfaces, and therefore the larger surface charge differences measured with respect to the control. This leads to a larger number of molecules attached to the surface, for both activated and nonactivated surfaces. On the other extreme, albumin-treated surfaces are only slightly less negative than the uncoated control surfaces. This is coherent with the inherently negative charge of this molecule at physiological pH, leading to a small change in the surface ζ potential.

**Table 2:** Isoelectric Points [pH­(I)] of the Proteins Used for the Functionalization of PETE

molecule	pH(I)^a^	charge at pH 7	Δpotential^b^
collagen	≈7.2 ^19^	neutral/positive	positive
gelatin	7–9.5 ^20^	neutral/positive	positive
albumin	4.7 ^21^	negative	positive
polylysine	≈9.2 ^22^	positive	positive

a Isoelectric point.

b Sample potential—control potential.

The apparently contradictory result that, based on the differences obtained, nonactivated surfaces promote better adhesion than the activated ones, could be explained by the differences in the protein binding mechanism between the nonactivated and activated surfaces, which affect the specific conformation of the protein at the surface (Figure 5).^23–29^ Indeed, nonactivated surfaces exert protein adsorption by electrostatic interactions between the negatively charged surface and the positively charged protein residues, while EDC/NHS activated surfaces anchor proteins by stronger covalent binding through amide bonds. In both cases, the protein molecules that remain at the interface are those that have strongly adhered to the surface. In nonactivated surfaces, the proteins that remain lay along the membrane surface due to electrostatic attraction (Figure 5A). In EDC/NHS activated surfaces proteins only need one strong covalent bond to remain anchored to the surface (Figure 5B). Therefore, the resulting mesh of proteins that remains on the surface and the actual extent of the protein coverage differ: nonactivated surfaces exerting long-range electrostatic forces may be fully coated, covered practically the whole surface with proteins, thus explaining to the larger change in ζ potential observed experimentally. In contrast, EDC/NHS activated surfaces offer stronger covalent binding due to the presence of activated esters, leading to less densely packed protein coatings. Therefore, more of the underlaying uncoated surface is still available, resulting in a smaller reduction of the ζ potential. Furthermore, due to these different protein conformations, the protein residues on activated surfaces are more available to interact with adhering cells than the proteins immobilized only through electrostatic interactions.^23,27–29^

**Figure 5:**
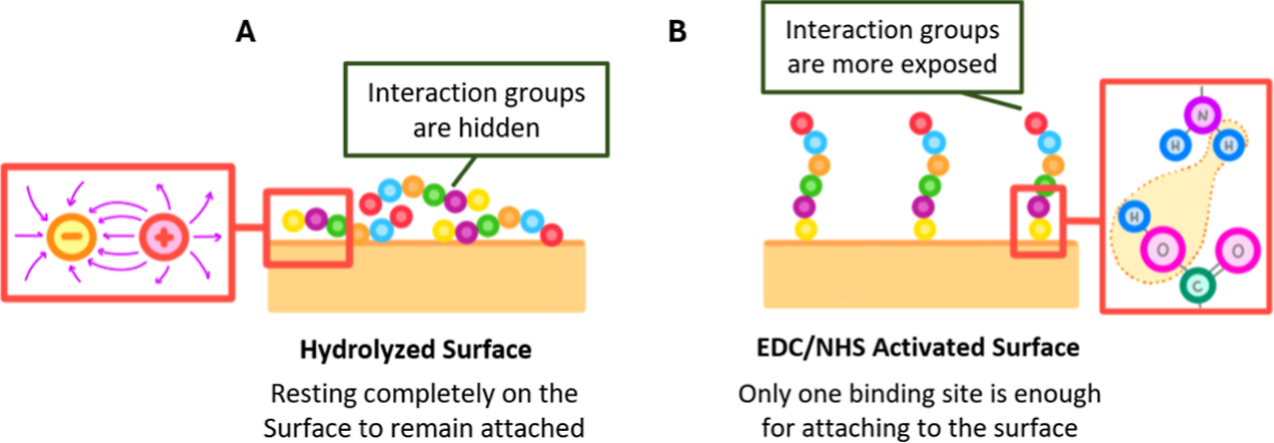
Hypothesis of the differential type of protein coating mechanisms for hydrolyzed and hydrolyzed and activated surfaces. Schematics showing protein conformational differences derived from surface functionality. (A) On hydrolyzed surfaces, the proteins lay on the membrane, producing less interactions with its surrounding media and efficiently screening the underlaying membrane surface charge. (B) On activated surfaces, one side of the protein chain covalently attaches to the surface, leaving the protein functional groups more exposed.

To complete the chemical characterization of the membranes, we measured the surface energy of the samples by quantifying the contact angle of the substrates in three different solvents: PBS, DI water, and glycerol. Specifically, we measured the dispersive energy and polar interaction components of the surface tension to dissect the different forces contributing to the surface energy. The results are shown in Figure 6.

**Figure 6:**
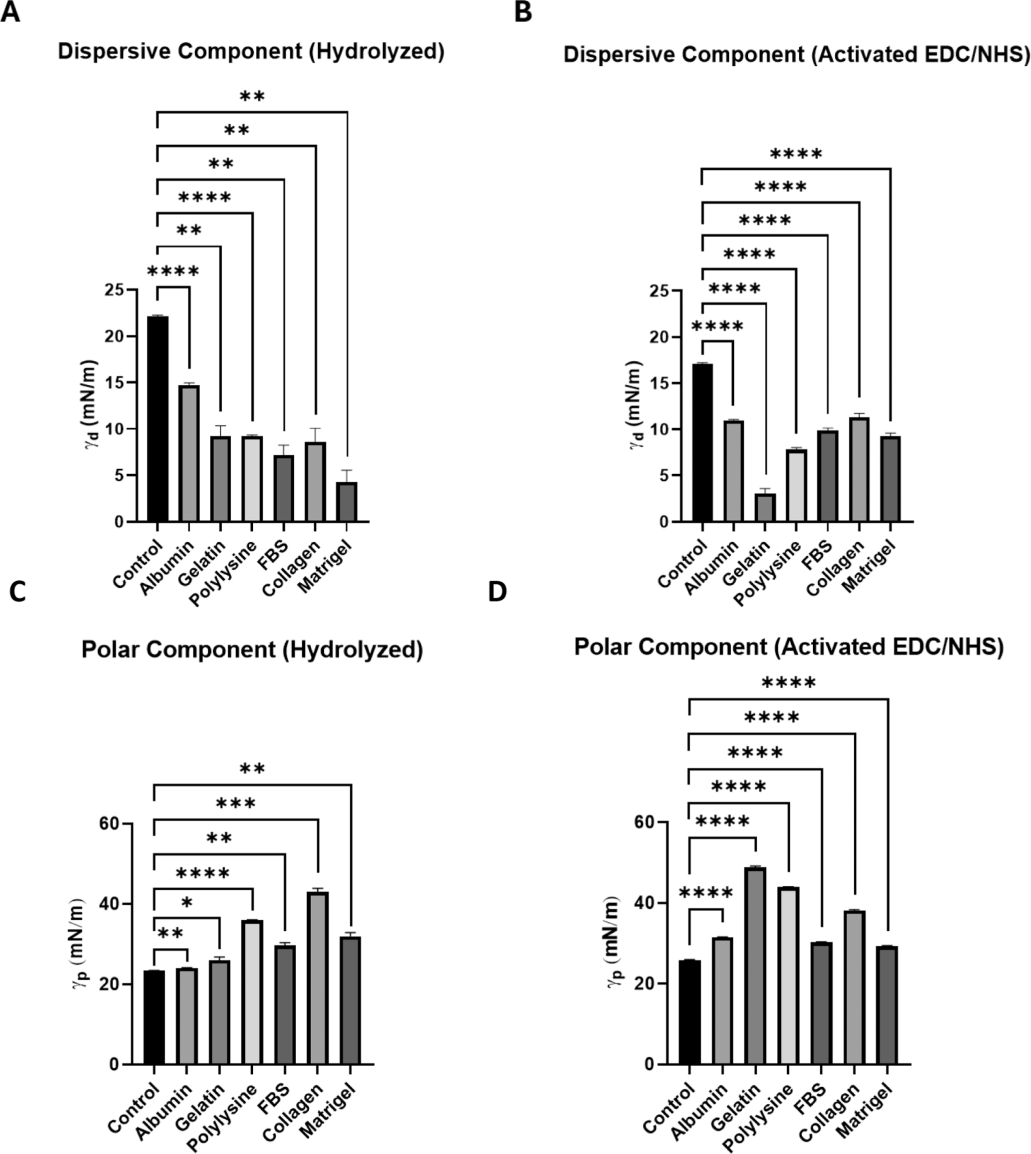
Surface energy measurement: dispersive and polar component. Dispersive component of the surface tension of (A) nonactivated and (B) activated treatments and polar component of the surface tension of (C) nonactivated and (D) activated surfaces. The graphs represent the media ± SD; *n* = 3 membranes [(A,C) unpaired two-tailed Welch tests, (B,D) unpaired two-tailed *t* tests].

The data indicate that both components of the surface energy in the protein-modified membranes differ from those of the controls. Functionalized substrates exhibit a lower dispersive component (Figure 6A,B) and a higher polar component (Figure 6C,D) compared to control PETE membranes, confirming surface modification in every treated sample, including FBS and Matrigel treated membranes, in which as mentioned before ζ potential measurements could not be obtained.

Organic polymers such as PETE are typically characterized by a dominant dispersive component and a lower polar component, which leads to a low wettability and minimal interactions with surrounding molecules.^30–33^ This dispersive component of surface tension is primarily related to van der Waals interactions, which are present in all material and are responsible for the attraction between nonpolar molecules. As protein molecules adhere to the surface, their polar functional moieties (like amine, carboxyl or hydroxyl groups) begin covering nonpolar areas of the subjacent substrate, reducing the dispersive component and enhancing the polar component of the surface. This is the case for all protein coatings tested experimentally. The polar component is associated with dipole–dipole interactions, hydrogen bonding, and other polar forces involving charged moieties, leading to increased wettability. In our case, this confirms the effectiveness of the protein coating achieved through our PETE surface functionalization protocol. Finally, the most significant changes in polar interaction component were observed in membranes treated with collagen, gelatin, and polylysine.

### 3.3 Cell Adhesion Assays on Modified Surfaces: Static Assays

We next evaluated cell adhesion to the coated surfaces under static conditions. To this end, we used the lung carcinomatous basal-alveolar immortalized epithelial cell line A549, and a hybrid cell line derived by the fusion of human umbilical vein endothelial cells (HUVECs) with the A549 cell line, EA.hy926, both commonly used to simulate the airway cellular composition in airway-on-chip devices. Both cell lines, GFP transfected, were cultured for 4 h on the PETE hydrolyzed membranes, protein-coated with and without EDC/NHS activation. The percentage of the area covered by the seeded cells on the whole region was quantitatively determined using automated image analysis, obtaining the results displayed in Figure 7.

**Figure 7:**
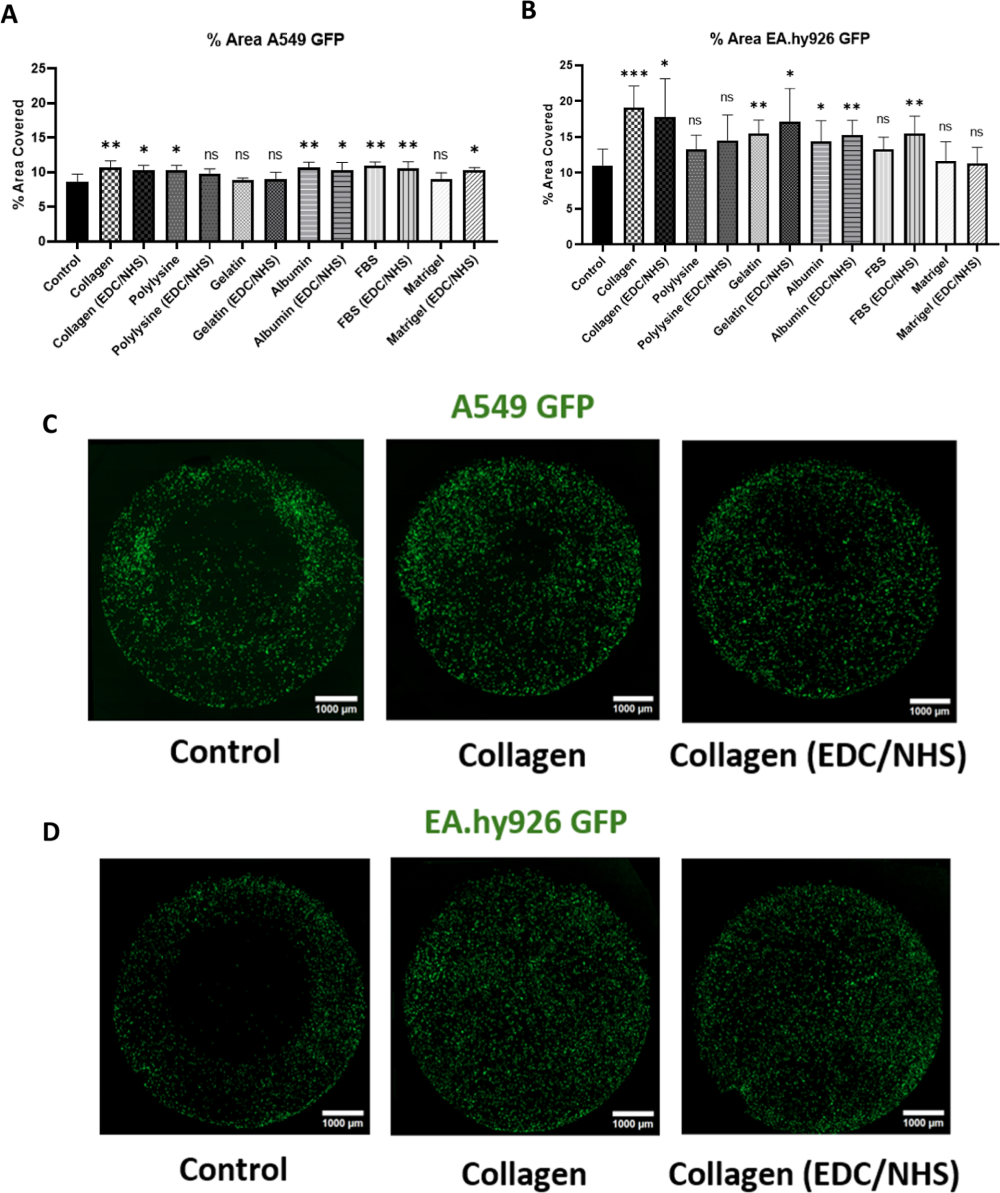
Analysis of cell adhesion under static conditions. Percentage of area covered by (A) A549 GFP and (B) EA.hy926 GFP cell lines on the different surfaces after a 4 h incubation. The graphs represent the mean ± SD; *n* = 6 (6 devices seeded with 6 independent cell cultures) (unpaired Welch tests with respect to the control). Representative images are displayed in (C) for A549 and (D) for EA.hy926 cell lines.

Globally, most surface treatments significatively enhance the adhesion of both cell lines used, with collagen-treated membranes displaying the best performance for both the cell lines investigated. Interestingly, as seen with the surface tension analysis, collagen-treated membranes displayed one of the largest increases on surface polar interactions. According to the literature, stronger cell adhesion to an underlying surface can be expected if the ratios of both components are similar between two interacting moieties.^34^ Therefore, the measured growth in the polar component of PETE membranes by the incorporation of protein molecules increases their wettability to a level similar to that of the cellular layer, thus improving cell attachment to the surface.

This is clearly demonstrated in the static adhesion experiments, where practically all the protein coatings tested improved cell adhesion. Additionally, from these results, it can be concluded, that the most suitable surfaces are the ones coated with type I collagen, as they provide a highly improved adhesion for both cell lines. Therefore, collagen-based surface treatments were used for the dynamic studies.

### 3.4 Cell Adhesion Assays on the Modified Surfaces: Dynamic Assay

To analyze cell attachment under dynamic conditions, four different surfaces were tested using the same two cell lines used in the static assays: untreated membranes (control), membranes treated with collagen via direct adsorption, which is the standard coating method used in the field,^9–11^ hydrolyzed surfaces treated with collagen, and hydrolyzed and activated surfaces treated with collagen. We assessed if the pretreatments performed on the membranes enhanced protein adhesion and, consequently, the attachment of the cells. The cells were seeded in micropatterned channels and incubated for 4 h. Then, low and moderate flow rates (see Table 1) were applied to each device to test cellular attachment under dynamic conditions. It is important to highlight that the lowest flows used for each cell line were selected following the experimental setups found in the literature, as described in Section 2. At low flow rates (Figure S3), A549 cells did not display a significantly improved cell attachment to hydrolyzed only or hydrolyzed and activated surfaces compared to the control surfaces or the surfaces treated with collagen via direct adsorption (standard treatment). In the case of EA.hy926 cells, all collagen treated surfaces led to improved cell coverage compared to the untreated surfaces, but no differences were found between hydrolyzed only or hydrolyzed and activated surfaces and the surfaces treated with collagen via direct adsorption (standard treatment).

Surface functionalization effects became clearer at moderate flow rates (Figure 8). When A549 and EA.hy926 cells were exposed to stronger shear stresses, differences between treatments showed higher adhesion for both hydrolyzed and activated surfaces. Hydrolysis of the membranes alone enhances cell adhesion compared to the control and the direct collagen (standard) treatment due to the increased interactions produced by the generated carboxylic groups. These improvements are even more pronounced when using the proposed EDC/NHS covalent activation treatment, resulting in better cell coverage than all the other treatments used. This result aligns with the initial hypothesis based on ζ potential measurements, confirming that the molecular conformation achieved through covalent bonding provides robust anchoring points for cell attachment even under higher flow rates. Summarizing, the combination of both hydrolyzation and activation demonstrated superior performance at high flow rates compared to the standard treatment used for these devices, being more significative for activated surfaces.

**Figure 8:**
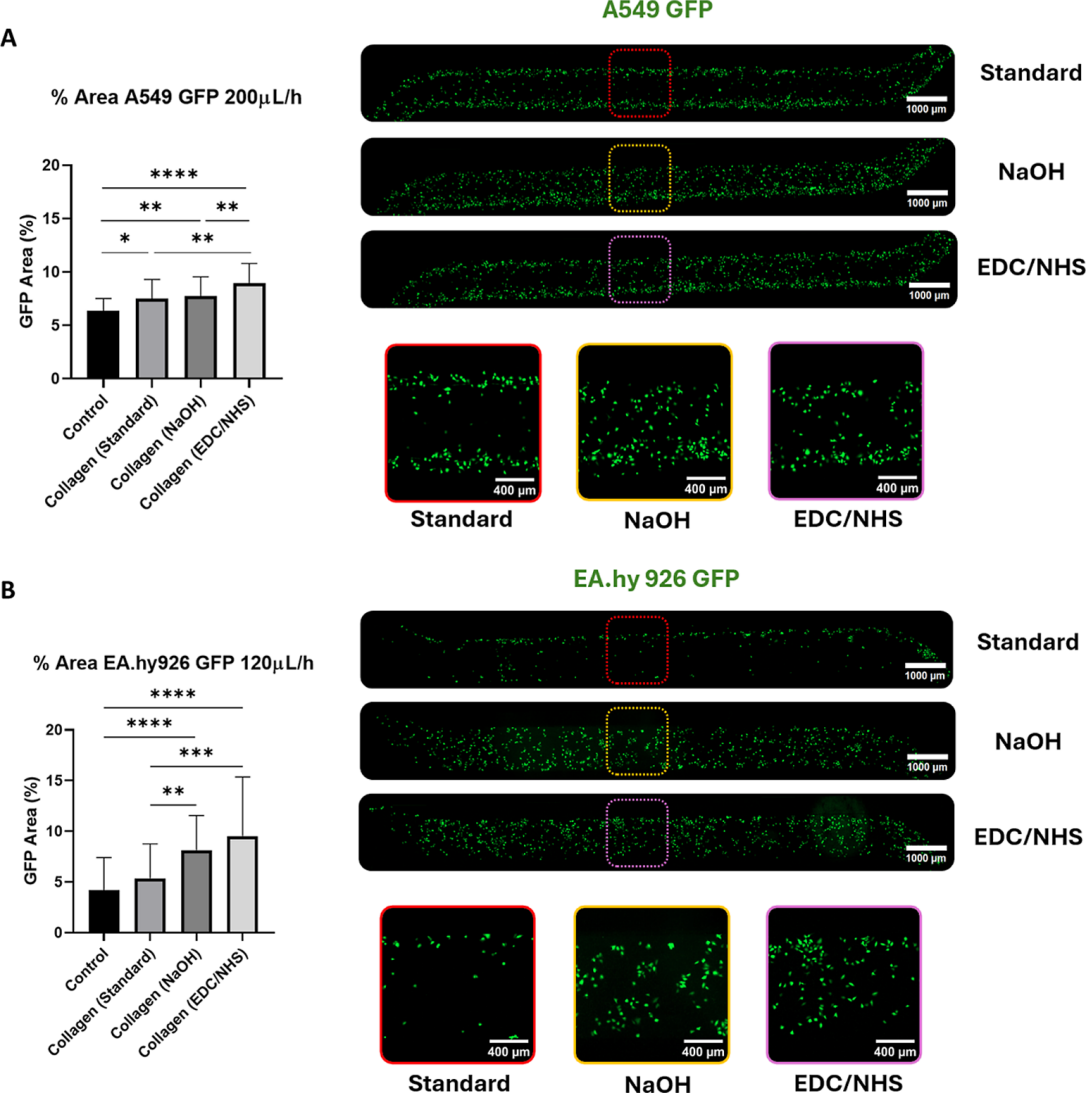
Analysis of cell adhesion under dynamic conditions: moderate flow rates. Percentage of area covered by GFP expressing cell lines [(A) A549, (B) EA.hy926] at medium flow rates. The graph represents the media ± SD; *n* = 40 (5 devices seeded with 5 independent cell cultures, 8 images per device) (Kruskal–Wallis test). Representative images are also displayed in (A) for A549 and (B) for EA.hy926 cell lines.

To further characterize the robustness of our best performing method, i.e., the combination of both hydrolyzation and activation of collagen, additional highest flows were tested to determine their capacity to retain cells at even more demanding conditions (see Table 1).

As shown in Figure 9, increasing the flow from low to moderate leads to detachment of cells from the modified surface, resulting in reduced coverage of the device. However, the decrease in the covered area is small, still supporting the robustness of the proposed treatment in withstanding higher flow rates. Furthermore, there seems to be a threshold for the initial detachment beyond which the treatments remain effective for those cells still attached to the surface. Indeed, no statistically significant differences are observed between devices subjected to intermediate and high flow rates, suggesting that cells not well adhered are removed under moderate flow conditions, while strongly adhered cells remain, resulting in similar outcomes even under more demanding conditions.

**Figure 9:**
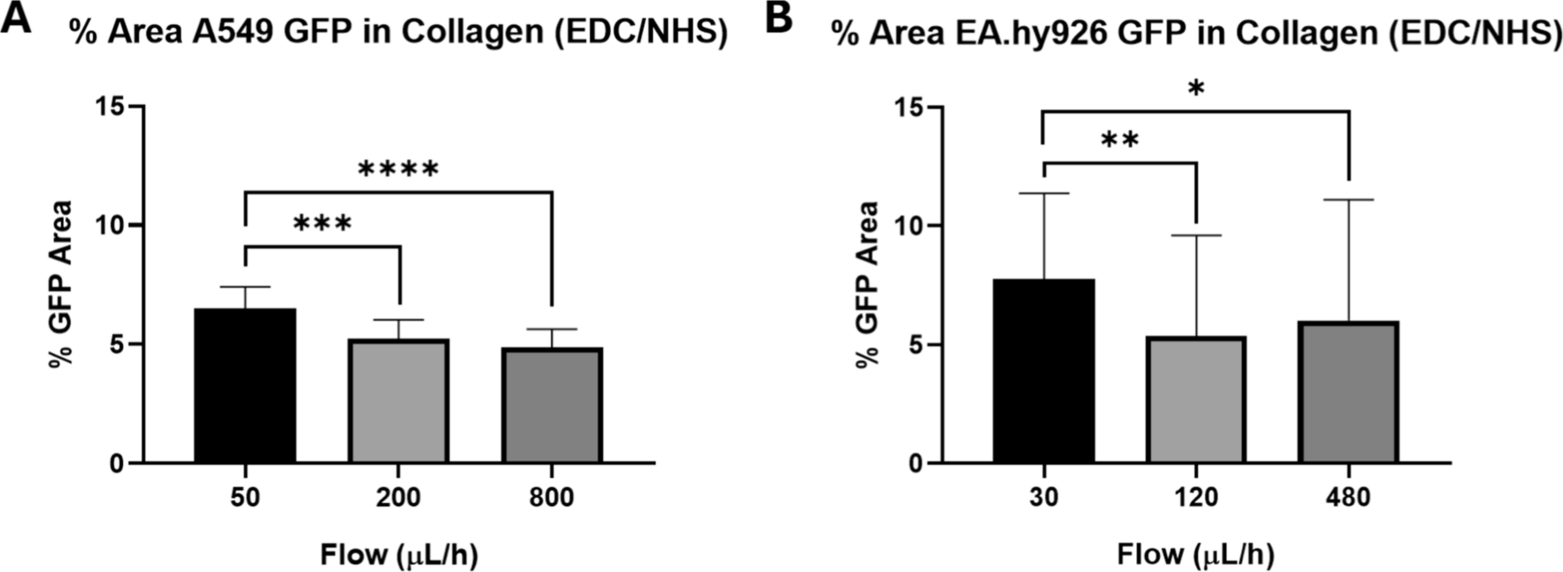
Analysis of cell adhesion for hydrolyzed and activated surfaces at increasing flow rates. Percentage of area covered by (A) A549 and (B) EA.hy926 GFP expressing cell lines at different flow rates in devices treated with hydrolyzed, EDC/NHS activated and coated with collagen. The graph represents the media ± SD. In (A), *n* = 16 (2 devices seeded with 2 independent cell cultures, 8 images per device) (ordinary one-way ANOVA, Tukey’s posthoc test). In (B), instead, *n* = 48 (6 devices seeded with 6 independent cell cultures, 8 images per device) (Kruskal–Wallis test, Dunn’s posthoc test).

### 3.5 Monolayer Formation under Dynamic Conditions on the Proposed Surface

Finally, we assessed the formation of a cellular monolayer on the modified PETE surface under dynamic conditions to show that the functionalization is suitable for OOC applications, allowing the formation of integral cellular tissues. For that, cells were incubated under dynamic conditions in the microfluidic microchannels, letting them adhere to the surface for 4 h and flowing media for 2 or 3 days, depending on the cell line. The epithelial devices were maintained for 2 days at a moderate flow of 200 μL h^–1^, whereas the endothelial devices were maintained for 3 days at the intermediate flow of 120 μL h^–1^. After that, devices were fixed and PECAM and E-Cadherin tight junctions were detected via immunofluorescence. The resulting images are shown in Figure 10 where it is observed that, at this flow conditions, the tight junction proteins are beginning to be expressed in some areas of the generated cellular layer, confirming that a cell barrier is being generated in the modified surface, allowing the formation of tissues in the PETE membrane.

**Figure 10:**
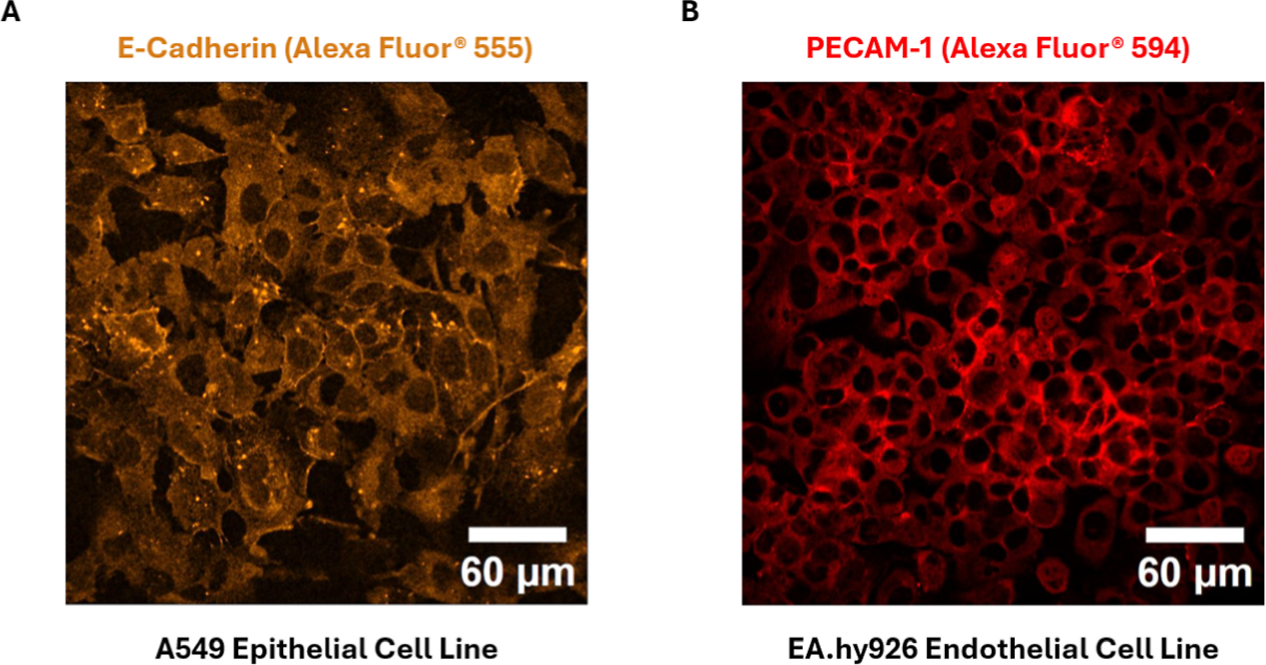
Formation of cellular layer under high-flow conditions. Confocal images obtained from the epithelial and endothelial devices after immunofluorescence staining. Panels (A,B) depict the expression of E-Cadherin and PECAM in the epithelial and endothelial channels, respectively, demonstrating monolayer formation under moderate flow rates.

## 4 Conclusions

A surface functionalization protocol has been developed to enhance surface–protein interactions via covalent bonds. While similar protocols have been used previously for covalently attaching proteins to various surfaces, for instance, in the functionalization of biosensors’ membranes and nanoparticles, this is, to the best of our knowledge the first application of this methodology not only for functionalizing PETE membranes but also for doing so in the context of multilayer OOC systems.^35,36^

The proposed functionalization does not affect the integrity of PETE membranes within OOC devices, while introducing desirable changes on surface energy. It is also remarkable that, with the proposed approach, robust immobilization of proteins can be obtained using reversible EDC/NHS activation without the need of complex, advanced chemicals such as functional silanes, thus leading to simpler and fully biocompatible systems, and relying on inexpensive reagents and standard laboratory equipment.^37,38^ As the method is based on amide bond formation it is broadly applicable and versatile. Indeed, it can be extended to any target protein for improving cell adhesion of specific cell lines.

Detailed chemical characterization of protein-coated surfaces allowed the determination of key properties affecting wettability and adhesion at these complex interfaces. Particularly, we observed an enhancement of cell coverage on collagen type I functionalized surfaces using two model cell lines. Our functionalization protocol also generates stable surface functionalities that improve resilience of two-dimensional cell cultures within OOC at higher flow rates compared to previously published systems, being possible to generate good cellular barriers even under these conditions and getting closer to the application of physiological flows in the devices. The findings from this work generate a set of surface treatments for PETE that not only lead to the development of more versatile devices in the OOC industry, but also to more reproducible and robust ones.

## Supplementary Material



## Data Availability

The raw/processed data required to reproduce these findings is available from the authors upon reasonable request.

## Notes

Additional experimental details, materials, and methods; working principle of UV–vis absorbance spectra algorithm, working principle of SEM image analysis algorithm, silica wafer micropatterning protocol; supplementary schemes; visual description of the main steps of the programmed algorithm for the analysis of the spectra obtained from the UV–vis spectrophotometer; supplementary figures; insert-device employed for carrying out static experiments in a more controlled way, microfluidic device employed for carrying out dynamic experiments, analysis of cell adhesion under dynamic conditions: low flow rates; supporting tables; heights of absorbance peaks (Δabsorbance) returned by the designed program in Matlab for each sample, heights of baselines (Δbaseline) returned by the designed program in Matlab for each sample (PDF)

## Abbreviations

PETE: polyethylene terephthalate
EDC: (3-(dimethylamino)­propyl) carbodiimide
NHS: *N*-hydroxysuccinimide
OOC: organ-on-a-chip
FDA: Food and Drug Administration
APPJ: atmospheric pressure plasma jet
PAC: plasma-activated coating
UV: ultraviolet
SEM: scanning electron microscopy
RT: room temperature
DI: deionized
MES: 2-(*N*-morpholino) ethanesulfonic acid
PTFE: Teflon
PBS: phosphate-buffered saline
BSA: bovine serum albumin
FBS: fetal bovine serum
PDMS: polydimethylsiloxane
PS: polystyrene
PMMA: poly­(methyl methacrylate)
PC: polycarbonate
COC: cyclic olefin copolymer
PLGA: poly­(lactic-*co*-glycolic)
COOH: carboxylic
Vis: visible
EHT: electron high tension
ppm: particles per million
DMEM: Dulbecco’s modified Eagle’s medium
DMSO: dimethyl sulfoxide
Pen/Strep: penicillin/streptomycin
EDTA: ethylenediaminetetraacetic acid
GFP: green fluorescent protein
CLAHE: contrast limited adaptive histogram equalization
PECAM: platelet endothelial cell adhesion molecule
FOV: field of view
SD: standard deviation.
