# Supplementary material for: Functionalization of Polyethylene Terephthalate (PETE) Membranes for the Enhancement of Cellular Adhesion in Organ-on-a-Chip Devices
Source: ACS Appl Mater Interfaces. 2025 Jan 8;17(3):4529–42. doi: 10.1021/acsami.4c17706 (PMC12123575; doi:10.1021/acsami.4c17706)
Supplement: Supplementary file 1 [file am4c17706_si_001.pdf]

Supporting Information

Functionalization of Polyethylene Terephthalate (PETE) Membranes for the Enhancement of Cellular Adhesion in Organ-on-a-Chip Devices

*Carlos Sobejano de la Merced [1,2], Lavinia Doveri [3], Tomás Muñoz Santoro [1,2], Javier García [1,2], Junkal Garmendia [4,5], Iván Cortés Domínguez [1,2†], Yuri Antonio Díaz Fernández[3†], and Carlos Ortiz de Solórzano [1,2,6† *]*

[1] University Clinic of Navarra Centre for Applied Medical Research, 31008, Pamplona, Spain

[2] University of Navarra Clinic Cancer Center, 31008, Pamplona, Spain

[3] Inorganic Nanochemistry Lab, University of Pavia, 27100, Pavia, Italy

[4] Instituto de Agrobiotecnología, 31192, Mutilva Baja, Spain

[5] CIBERES, 28029, Madrid, Spain

[6] CIBERONC, 28029, Madrid, Spain

[†] These authors contributed equally to this work

* Corresponding author. Email: codesolorzano@unav.es

1. Supporting Materials and Methods

1.1. Working Principle of UV-Vis Absorbance Spectra Algorithm. This algorithm has been designed to analyze and compare quantitatively the spectra coming from the UV-Vis spectrophotometer. The program first removes noise from the original signal (Supplementary Scheme S2.1.) by applying a Butterworth filter (order 1, cutoff frequency 0.3, lowpass filter). Next, the algorithm eliminates the diffraction pattern produced by the pores of the membrane, calculating first the envelope functions surrounding the original graph, and applying the mean between both, obtaining a clear line that allows performing comparisons between measurements (Supplementary Figure 1B). Then, a baseline is generated for each measurement in the 520 – 700 nm range, where the absorbance peak of the malachite green appears (Supplementary Figure 1C). This is done by interpolating between the absorbance values in the end points. By subtracting the original peaks with this baseline, their height (Δ Absorbance) can be obtained and compared (Scheme 2D). Additionally, the baselines for each sample can be compared, which are related to the opacity of the resulting etched surfaces (Supplementary Figure 1C). The comparison is made by subtracting the control baseline to the sample baseline (Δ Baseline).

1.2. Working Principle of Scanning Electron Microscopy (SEM) Image Analysis Algorithm. This algorithm allows the determination of polyethylene terephthalate (PETE) membranes' pore diameter, circularity and porosity through the analysis of SEM images obtained of the modified

surfaces. Briefly, the images were despeckled using a 3 x 3 median filter. Thresholding was then applied to the images using the default method available in the program, which is a variation of the IsoData algorithm.[1] It divides the image into objects and background by taking an initial threshold, then the averages of the pixels at or below the threshold and pixels above are computed. The averages of those two values are computed, the threshold is incremented, and the process is repeated until the threshold is larger than the composite average. 1 M samples, were thresholded using Yen's method due to its superior performance in segmenting images for these substrates compared to the default method described above.[2,3] Yen's method determines the optimal threshold value based on maximizing the sum of two terms that represent the between-class variance and the entropy of the image histogram. After segmentation, the pores' dimensions were measured using Fiji's "Analyze Particles" function, setting 0.1 μm as the minimum size of the pores and 0.5 as the minimum circularity of the pores. From this function, each pore's area and circularity was obtained. Additionally, the total number of pores in each image can be obtained from it.

1.3. Silica Wafer Micropatterning: Protocol. A mold for the 0.2 mm width x 1 mm height endothelial channel consisted of a patterned silica wafer fabricated by photolithography. First, a 4-inches silica wafer was exposed to a 1-minute oxygen plasma treatment at 85W (Diener Zepto, Germany). Then, 4 mL of SU8-2100 resin (Kayaku, MA, USA) were poured on the treated surface and spin coated in a SPIN150i-NPP (SPS, UK) following the next program: 500 revolutions per minute (RPM) with an acceleration of 100 RPM s^{-1} for 10 seconds and 1500 RPM with an acceleration of 300 RPM s^{-1} for 30 seconds. Next, the wafer was soft baked for 7 minutes at 65°C and 39 minutes at 95°C. After that, the resin was exposed to ultraviolet (UV) light (315 mJ cm^{-2}),

using a photolithography mask with the desired pattern (Micro Lithography Services Limited). Then, the wafer was hard baked for 5 minutes at 65°C and 14 minutes at 95°C. Finally, the wafer was submerged in SU8-Developer (Microchem) for 16 minutes, getting the desired pattern on the surface.

2. Supporting Schemes

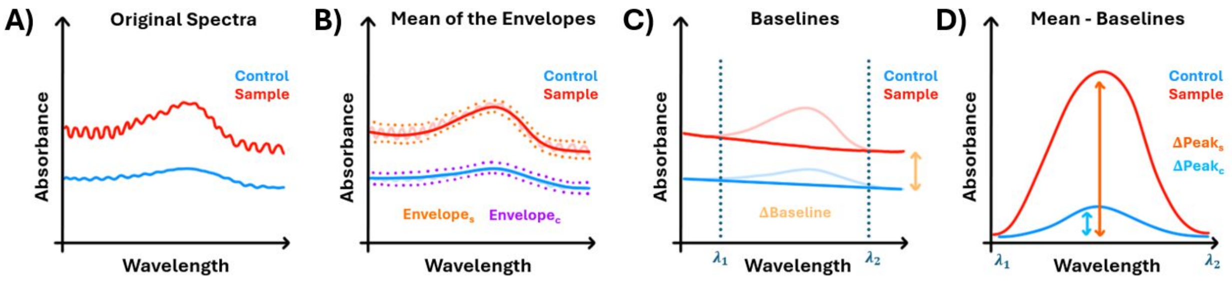

Scheme S1. Visual description of the main steps of the programmed algorithm for the analysis of the spectra obtained from the UV-vis spectrophotometer. (A) First, the envelopes are estimated, and (B) the mean is plotted. (C) Then, the baselines of the different samples are determined. With these values, the opacity of the membranes can be assessed (Δ Baseline). (D) By subtracting the baselines to the mean of the envelopes, the absorbance peaks in the range $[\lambda_1,\lambda_2]$ can be determined and compared (Δ Peak).

3. Supporting Figures

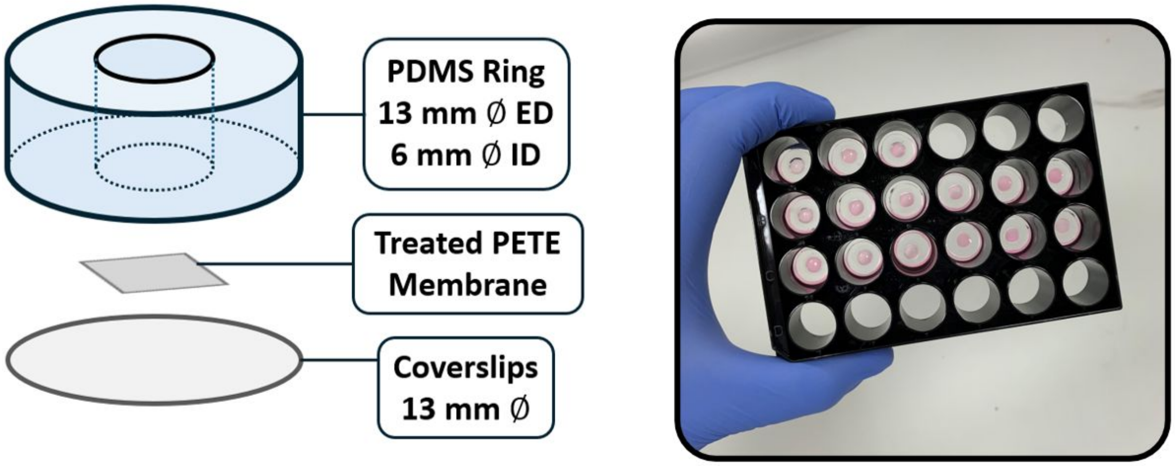

Figure S1. Insert-device employed for carrying out static experiments in a more controlled way. The device is composed of a polydimethylsiloxane (PDMS) ring, a coverslip and the studied membrane. The membrane is embedded in the system via plasma bonding between the other two elements. The final device is attached to a 24 well-plate by placing a thin film of uncured PDMS with curing agent.

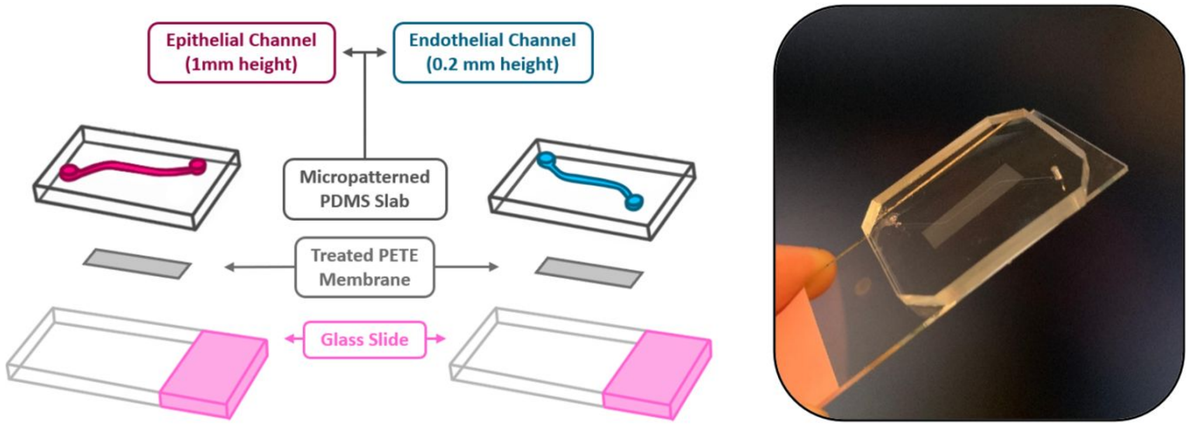

Figure S2. Microfluidic device employed for carrying out dynamic experiments. The device is composed of a PDMS slab which includes a micropatterned channel, a glass slide / coverslip and

the studied membrane. The membrane is embedded in the system via plasma bonding between the other two elements.

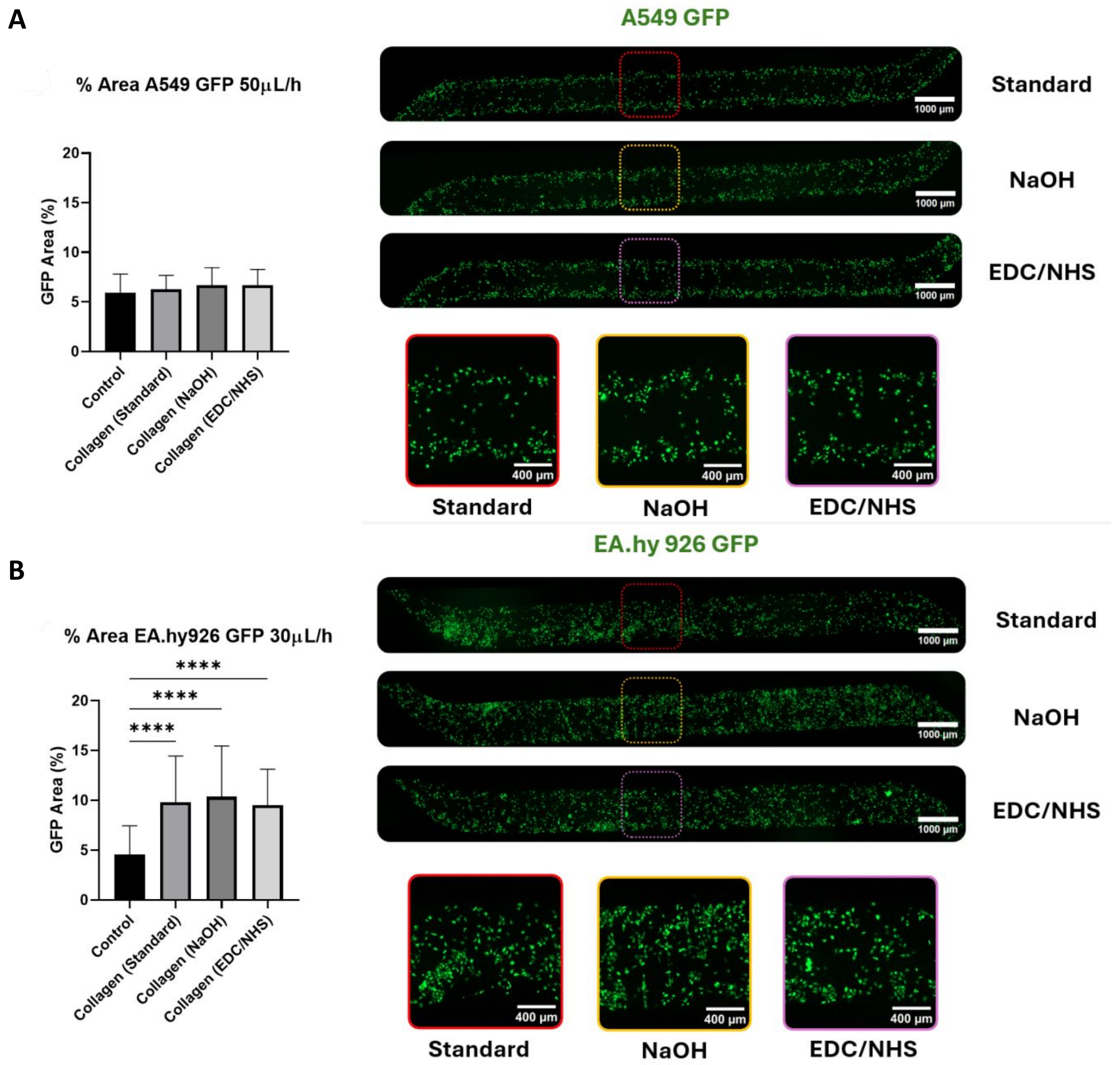

Figure S3. Analysis of cell adhesion under dynamic conditions: low flow rates. Percentage of area covered by green fluorescent protein (GFP) expressing cell lines (A A549, B EA.hy926) at low flow rates. The graph represents the media ± S.D.; $n = 40$ (5 devices seeded with 5 independent cell cultures, 8 images per device) (A Brown-Forsythe and Welch ANOVA, B Kruskal-Wallis test). Representative images are also displayed in A) for A549 and B) for EA.hy926 cell lines.

4. Supporting Tables

Table S1. Heights of absorbance peaks (Δ Absorbance) returned by the designed program in Matlab® for each sample.

Control	NaOH 0.25 M	NaOH 0.5 M	NaOH 1 M
0.0251	0.0568	0.0649	0.0470
0.0235	0.0506	0.0492	0.0408
0.0357	0.0622	0.0450	0.0369
0.0295	0.0548	0.0490	0.0544
0.0243	0.0526	0.0487	0.0397
0.0216	0.0572	0.0433	0.0394

Table S2. Heights of baselines (Δ Baseline) returned by the designed program in Matlab® for each sample.

Control	0.25 M	0.5 M	1 M
0.0037	0.0222	0.0799	0.2189
0.0111	0.0103	0.0596	0.1938
0.0012	0.0190	0.0649	0.2023
0.0044	0.0106	0.0538	0.1780
-0.0029	0.0131	0.0519	0.1966
0.0066	0.0225	0.0625	0.1903
0.0140	0.0096	0.0725	0.2115
0.0041	0.0148	0.0522	0.1864
0.0073	0.0029	0.0575	0.1949
0.0029	0.0116	0.0464	0.1707
0.0074	0.0032	0.0445	0.1892
-0.0025	0.0057	0.0551	0.1829
0.0007	0.0151	0.0823	0.2213
-0.0066	0.0022	0.0620	0.1962
-0.0037	0.0247	0.0674	0.2048

Control	0.25 M	0.5 M	1 M
-0.0074	0.0128	0.0562	0.1805
-0.0098	0.0214	0.0544	0.1990
-0.0067	0.0131	0.0649	0.1928
-0.0140	0.0155	0.0792	0.2182
-0.0111	0.0249	0.0589	0.1930
0.0025	0.0120	0.0642	0.2016
0.0098	0.0215	0.0531	0.1773
0.0032	0.0096	0.0512	0.1959
-0.0041	0.0183	0.0618	0.1896
-0.0012	0.0099	0.0864	0.2254
-0.0007	0.0124	0.0661	0.2003
0.0067	0.0217	0.0715	0.2089
-0.0032	0.0089	0.0603	0.1846
-0.0073	0.0288	0.0585	0.2031
-0.0044	0.0169	0.0690	0.1969

5. Supporting Bibliography

(1) Ridler, T. W.; Calvard, S. PICTURE THRESHOLDING USING AN ITERATIVE SLECTION METHOD. *IEEE Trans Syst Man Cybern* 1978, *SMC-8* (8), 630–632. https://doi.org/10.1109/TSMC.1978.4310039.

(2) Yen, J. C.; Chang, F. J.; Chang, S. A New Criterion for Automatic Multilevel Thresholding. *IEEE Transactions on Image Processing* 1995, *4* (3), 370–378. https://doi.org/10.1109/83.366472.

(3) Sankur, B. Survey over Image Thresholding Techniques and Quantitative Performance Evaluation. *J Electron Imaging* 2004, *13* (1), 146. https://doi.org/10.1117/1.1631315.
